# Validity and Reliability of an Estimated Daily Intake Scale for Fat

**DOI:** 10.5539/gjhs.v4n2p36

**Published:** 2012-03-01

**Authors:** Gregory J. Privitera, Chanel S. Freeman

**Affiliations:** Department of Psychology, Saint Bonaventure University 3261 West State Street, St. Bonaventure, New York, 14778 Tel: 716-375-2488 E-mail: gprivite@sbu.edu; St. Bonaventure University

**Keywords:** EDIS-F, Fat, Taste preference, Reliability, Validity

## Abstract

The reliability and validity of an Estimated Daily Intake Scale for Fat (EDIS-F) used to measure daily intake of fat in a participant’s diet was tested. A Cronbach’s alpha was used to determine the reliability of the EDIS-F. To determine the validity of this scale, scores on this scale were correlated with known factors related to daily intake of fat (e.g., ratings of liking for a high fat food and the body mass index score of participants). A 13-item EDIS-F was shown to be reliable, and scores on the EDIS-F significantly correlated with ratings of liking for a high fat cream cheese cracker and BMI, but not with gender, consistent with the assertion that this scale measures daily intake of fat. Implications for using this scale are discussed.

## 1. Introduction

Many areas of research are interested in fat intake, particularly those areas that investigate diet, health, taste preferences, and perception (Privitera, 2008; Rothblum, Solovay, & Wann, 2009; Veldhuizen, Rudenga, & Small, 2010). At present, no scale exists to measure the exposure participants have with a high fat diet, although some scales related to fat intake do have limited uses. These scales include the Fat Preference Questionnaire^©^ (Ledikwe, Ello-Martin, Pelkman, *et al.*, 2007), and daily or weekly diary scales such as the Medical Research Council 4-day diet diary (MRC-DD; Cambridge, United Kingdom). While fat intake is related to preferences for high fat foods (Drewnowski & Hann, 1999), the first measure is primarily a measure of preference and diet diaries can take days or weeks to complete. A brief assessment of fat intake would be useful for researchers to quickly measure fat intake, which could have many potential applications.

A brief scale that measures daily fat intake could be used to group participants based upon their levels of exposure to high fat foods, or could control for fat intake as a covariate. Such a scale could also be used to quickly measure fat intake as a factor of interest. For example, recent brain imaging studies show that displaying pictures of high calorie foods can increase activity in brain reward regions (Frank *et al.*, 2010); other studies show that high fat diet intake can lead to learning deficits (Privitera, Zavala, Sanabria, & Sotak, 2011), and influence flavor preference learning (Capaldi & Privitera, 2007). A measure for the amount of fat in a participant’s diet could be used to determine how high fat diet intake is related to these changes in brain activity and discrepancies in learning using human participants. If used with the estimated daily intake scale for sugar (EDIS-S; Privitera & Wallace, 2011), researchers could more readily investigate how the intake of sugars and fats (included as quasi-independent factors for human studies) differentially influence any number of factors of interest.

In sensory conditioning studies, sugars are often used as taste unconditioned stimulus (US) and fat is used as a nutrient US. Prior to conducting studies using a sugar US, researchers can use the EDIS-S to determine which participants like sugar, and will therefore regard sugar as a US (Privitera *et al.*, 2011). Participants scoring a 39 or higher on the EDIS-S are selected for these studies because this score corresponds to a rating of at least 50 mm for sugar water on a 100 mm rating scale. Similarly, it would be advantageous to have a scale that is related to liking for high fat foods by determining what minimum score on such a scale corresponds to a rating of at least 50 mm for a high fat food on a 100 mm scale. This would allow researchers to use such a scale to select participants for studies that use fat as a nutrient US in the same way that the EDIS-S is used to select participants when sugar is used as a US.

The aim of our study was to construct a brief scale, called the Estimated Daily Intake Scale for Fat (EDIS-F), to measure a participant’s intake of fat in their diet. Such as scale has not been previously used in published research, and was adapted from the EDIS-S (Privitera *et al.*, 2011) by developing 24 statements about the extent to which participants include fat in their diet. Participants indicated their level of agreement to each statement on a 7-point Likert scale in which higher scores indicated greater intake of fat. It was expected that scores on the EDIS-F would be related to liking ratings for a high fat food and BMI, but not related to gender. The more exposure participants have with fat in their diets, the more they should like a high fat food (Birch & Marlin, 1982; Mennella & Beauchamp, 1996; 2002; Pliner, 1982; Privitera, 2008), and BMI is associated with risk factors of obesity and so should be related to fat intake (World Health Organization, 2012). Also, while men tend to consume more fat (g) than women, the percent of total energy consumed of fat shows marginal gender differences (Kennedy, Bowman, & Powell, 1999). For this reason, total fat intake in relation to total daily intake should not vary by gender, and so we also tested if scores on the EDIS-F were related to gender differences to establish discriminant validity of the EDIS-F.

## 2. Method

### 2.1 Participants

A convenience sample of 110 participants (65 women, 45 men) was recruited to participate in this study from a university in the northeast. Participant characteristics (*M*±SD) were as follows: age (19.8±1.8 years), weight (162.0±21.4 lbs), height (67.5±3.0 in), and Body Mass Index (24.6±4.2 kg/m^2^). Participants were non-smokers and in general good health with no participants reporting physical or doctor diagnosed food allergies, medical conditions including pregnancy, or dietary restrictions, as determined in a prescreening session. Because women with a history of dieting can be insensitive to flavor-based learning (Brunstrom, Downes, & Higgs, 2001), participants scoring 9 or higher on the Restraint Scale of the Three Factor Eating Questionnaire (Stunkard & Messick, 1985) were excluded. Hunger states can also influence learning when a caloric US is used (Capaldi *et al.*, 1994; Fedorchak & Bolles, 1987; Yeomans & Mobini, 2006), so participants who ate within two hours of the study were also excluded. All procedures were approved by the university’s Institutional Review Board.

### 2.2 Procedures

Participants were observed in a classroom setting for 25 to 30 minutes between 10:00AM and 1:00PM in an experimental session. All participants were instructed not to talk to other participants and to look ahead, and were run through a preliminary phase and a tasting phase during the session.

*Preliminary phase*. Participants signed an informed consent and were told to complete a packet of written questionnaires. The questionnaires included general demographic questions and included 24-items for the EDIS-F, in which participants indicated their level of agreement for each item on the EDIS-F on a 7-point rating scale ranging from 1 (*completely disagree*) to 7 (*completely agree*). Once the questionnaire was completed, participants were told to flip the packet upside down on their table and wait for further instructions.

*Tasting phase*. Immediately following the preliminary phase, participants were asked to taste and rate one high fat cream cheese cracker spread. Participants were asked to eat the cracker, and then rate it on a 100 mm line ranging from 0 mm (*very unpleasant*) to 100 mm (*very pleasant*). The high fat Philadelphia brand cream cheese was spread onto a small cracker and was about 40 calories and 2 g of fat per cracker. The cream cheese cracker was given on a 5 in paper plate and served with water given in a 3 oz plain Dixie paper cup. Once the tasting phase was complete, participants were debriefed, thanked for their time, and dismissed.

### 2.3 Statistical Analyses

IBM SPSS® Version 19.0 was used to analyze the data. A Cronbach’s alpha was computed for the 24-item EDIS-F to measure the reliability of the EDIS-F. The number of items included in the survey was reduced to maximize the alpha coefficient. Items on the scale were removed one item at a time. The item removed each time was the item that increased the value of the alpha coefficient the most, if removed. Items were removed until removing any further items would decrease the value of the alpha coefficient. To determine convergent validity, a Pearson correlation was computed for scores on the final EDIS-F (i.e., the final version that maximized the alpha coefficient), pleasantness ratings for the high fat cream cheese crackers (rated in the tasting phase), and BMI. To establish discriminant validity, a point-biserial correlation between gender and scores on the EDIS-F was also computed with gender as the dichotomous factor and EDIS-F scores as the continuous factor.

To determine which minimum score on the EDIS-F corresponds to a liking rating of 50 mm or higher on the 100 mm rating scale for the high fat food, the method of least squares was used. Using this analysis, EDIS-F scores were the criterion variable and pleasantness ratings for the high fat food were the predictor variable. A rating of 50 mm was entered into the equation of the regression line to determine the score on the EDIS-F (the criterion variable) that corresponded to a 50 mm rating for the high fat food.

## 3. Results

*Internal consistency reliability*. A Cronbach’s alpha reliability coefficient was. 876 for the 24-item EDIS-F scale. The 24 items were removed using the same criteria described in the statistical analyses section. The final scale was a 13-item EDIS-F with a large alpha coefficient of. 920. Table 1 lists the mean, standard deviation, and corrected item-total correlation for each item on the final EDIS-F. On all 13 items, scores were 50.83±16.29 (*M*±SD) on the EDIS-F. The unreliable items that were removed from the original list of items are given in Table 2.

**Table 1:** The mean, standard deviation, and corrected item-total correlation for the reliable 13-item EDIS-F. An asterisk (*) indicates items in which ratings were reversed. These items were included to avoid a response set pitfall. To score reversed items, flip a participants’ score before adding the total score

Item	Statement	Mean	Standard Deviation	Item-Total Correlation
1	I tend to enjoy eating high fat flavorful foods in a meal.	4.28	1.79	0.53
2*	I tend to eat foods that are low fat, even desserts.	4.52	1.52	0.63
3	I tend to snack on cakes, cookies, or brownies when I am hungry.	2.99	1.81	0.60
4	I tend to crave foods that are high in fat.	4.12	1.78	0.66
5	I tend to eat high fat meals each day.	3.84	1.67	0.72
6*	I tend to snack on healthier, low fat food options.	3.79	1.7	0.77
7*	I generally tend to consume a low fat diet.	4.15	1.75	0.74
8*	I tend to avoid desserts that are too fattening.	4.39	1.79	0.60
9	When I crave a snack, I typically seek out high fat foods.	3.24	1.68	0.66
10	I tend to eat fast foods, especially when I am in a rush.	3.75	2.16	0.67
11	I like consuming high fat foods that are flavorful.	3.65	1.60	0.66
12	I typically will eat a snack, even when only high fat foods are available.	4.51	1.74	0.54
13	I generally tend to enjoy consuming a high fat diet.	3.59	1.78	0.77

**Table 2:** Removed items from the original 24-item list. These items were removed to maximize the value of the Cronbach’s alpha reliability coefficient for the scale

Removed Item	Statement
1	I often put butter on rice, noodles, potatoes, and/or other foods in a meal.
2	I often eat flavored chips (such as Doritos or Cheez-Its) to snack on.
3	I will eat less in a meal to “save room” for a high fat dessert.
4	I tend to add oil or mayonnaise to add flavor to a sandwich.
5	I often crave fatty foods after I just finished a meal.
6	I tend to spread butter on toast, bagels, pancakes or other related breakfast foods.
7	I often put cheeses or spreads on my vegetables in a meal.
8	I typically will snack on vegetables when I can dip them in spreads (such as blue cheese or ranch dressing).
9	I will eat a high fat dessert before my meal if given the opportunity.
10	I tend to eat a lot of vegetables and salads in a meal.
11	When I am full after a meal, I usually “have room” for a high fat dessert.

*Correlation analyses*. To determine the validity of the EDIS-F as a measure of exposure to high fat foods, the correlation between total scores on the 13-item EDIS-F, BMI scores, gender, and 100 mm ratings for the high fat cream cheese crackers was computed. As expected, scores on the 13-item EDIS-F were positively correlated with 100 mm ratings for the high fat cream cheese cracker, *r* = .62, *p* < .001, and BMI scores, *r* = .34, *p* < .001, but were not related to gender, *r*_pb_ = .11, *p* = .26.

**Figure 1:**
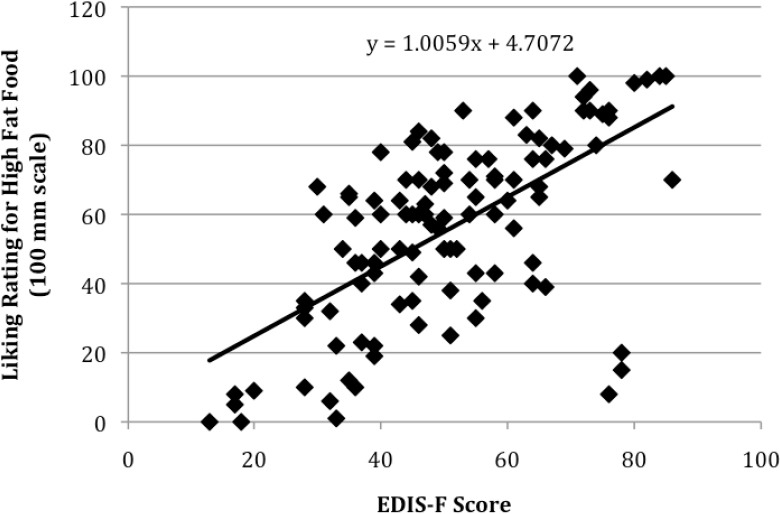
The data points and regression line for the relationship between scores on the 13-item EDIS-F and 100 mm ratings for the high fat food

*Computing cutoff scores*. Using the method of least squares, the 13-item EDIS-F scores were the criterion variable and pleasantness ratings for the high fat food were the predictor variable. As illustrated in Figure 1, the equation of the regression line for the relationship between these two factors was *Y* = 1.0059*X* + 4.7072, = .38. When *X* = 50 mm rating for the high fat food, the corresponding value was *Y* = 55.00. Hence, a score of 55 on the EDIS-F corresponds to a 50 mm rating for the high fat food.

## 4. Discussion

The aim of this study was to determine the reliability and validity of a scale that could be scored quickly and used to estimate daily fat intake without requiring participants to consume a high fat food. Participants completed the EDIS-F and also tasted and rated a high fat cream cheese cracker on a 100 mm scale. The results showed that a 13-item EDIS-F was a very reliable scale. The scale also met two criteria for validity in that scores on EDIS-F were, as expected, positively correlated with ratings of liking for the high fat food and BMI (convergent validity), but not related to gender (discriminant validity). These findings suggest that the EDIS-F is a measure for how much fat participants consume in their diets, with higher scores indicating higher intakes of fat.

Moreover, a regression analysis showed that a minimum score of 55 on the EDIS-F corresponds to a rating of at least 50 mm on a 100 mm pleasantness (liking) scale for a high fat food. This criterion can be used to select participants for conditioning studies that use a high fat food as a US to ensure that only participants who perceive fat as a US are included in such studies. Hence, using the EDIS-F, participants scoring 55 or higher can be included; those scoring less than 55 can be excluded from participating in conditioning studies that use a high fat food as a US.

A key limitation of the EDIS-F is that it is unknown as to whether such a scale could be used to predict future health outcomes. Such a possibility, however, is testable and so future studies can test this possibility using research designs that are longitudinal and cross-sectional. At present, the EDIS-F provides a reliable and valid measure that can be used across disciplines to account for fat intake as a factor of interest or as a potential covariate. This scale can also be used as a participant selection tool for conditioning studies that use fat as a US, in the same way that the EDIS-S is currently used as a participant selection tool when sugar is used as a US. In all, the EDIS-F can be a reliable and valid tool for researchers interested in fat intake across disciplines, including but not limited to research areas related to diet, health, learning, sensation, and taste perception.
